# Chromosomal distribution of interstitial telomeric sequences as signs of evolution through chromosome fusion in six species of the giant water bugs (Hemiptera, *Belostoma*)

**DOI:** 10.1002/ece3.3098

**Published:** 2017-06-07

**Authors:** Mónica G. Chirino, Martina Dalíková, František R. Marec, María J. Bressa

**Affiliations:** ^1^ Grupo de Citogenética de Insectos Instituto de Ecología, Genética y Evolución de Buenos Aires Departamento de Ecología, Genética y Evolución, Facultad de Ciencias Exactas y Naturales Universidad de Buenos Aires Ciudad Autónoma de Buenos Aires Argentina; ^2^ Consejo Nacional de Investigaciones Científicas y Técnicas Ciudad Autónoma de Buenos Aires Argentina; ^3^ Laboratory of Molecular Cytogenetics Institute of Entomology Biology Centre ASCR České Budějovice Czech Republic

**Keywords:** chromosomal fusion, interstitial telomeric repeats, karyotype evolution, telomere FISH

## Abstract

Tandem arrays of TTAGG repeats show a highly conserved location at the telomeres across the phylogenetic tree of arthropods. In giant water bugs *Belostoma*, the chromosome number changed during speciation by fragmentation of the single ancestral X chromosome, resulting in a multiple sex chromosome system. Several autosome–autosome fusions and a fusion between the sex chromosome pair and an autosome pair resulted in the reduced number in several species. We mapped the distribution of telomeric sequences and interstitial telomeric sequences (ITSs) in *Belostoma candidulum* (2n = 12 + XY/XX; male/female), *B. dentatum* (2n = 26 + X_1_X_2_Y/X_1_X_1_X_2_X_2_), *B. elegans* (2n = 26 + X_1_X_2_Y/X_1_X_1_X_2_X_2_), *B. elongatum* (2n = 26 + X_1_X_2_Y/X_1_X_1_X_2_X_2_), *B. micantulum* (2n = 14 + XY/XX), and *B. oxyurum* (2n = 6 + XY/XX) by FISH with the (TTAGG)_*n*_ probes. Hybridization signals confirmed the presence of TTAGG repeats in the telomeres of all species examined. The three species with reduced chromosome numbers showed additional hybridization signals in interstitial positions, indicating the occurrence of ITS. From the comparison of all species here analyzed, we observed inverse relationships between chromosome number and chromosome size, and between presence/absence of ITS and chromosome number. The ITS distribution between these closely related species supports the hypothesis that several telomere–telomere fusions of the chromosomes from an ancestral diploid chromosome number 2n = 26 + XY/XX played a major role in the karyotype evolution of *Belostoma*. Consequently, our study provide valuable features that can be used to understand the karyotype evolution, may contribute to a better understanding of taxonomic relationships, and also elucidate the high plasticity of nuclear genomes at the chromosomal level during the speciation processes.

## 1 INTRODUCTION

Telomeres are specialized nucleoprotein complexes localized at the ends of linear eukaryotic chromosomes. They maintain the stability and integrity of the chromosomes by protecting their ends from the action of exonucleases, end‐to‐end fusions, and gradual erosion during successive rounds of semiconservative DNA replication (Blackburn, 1991; Fajkus, Sýkorová, & Leitch, 2005; de Lange, 2004; Louis & Vershinin, 2005). In most eukaryotes, telomeric DNA is composed of long arrays of a short repetitive sequence. This also applies to arthropods with the ancestral and most common telomeric motif (TTAGG)_*n*_ (Frydrychová, Grossmann, Trubač, Vítková, & Marec, 2004; Korandová, Krůček, Vrbová, & Frydrychová, 2014; Lorite, Carrillo, & Palomeque, 2002; Okazaki, Tsuchida, Maekawa, Ishiikawa, & Fujiwara, 1993; Sahara, Marec, & Traut, 1999; Traut et al., 2007; Vítková, Král, Traut, Zrzavý, & Marec, 2005). However, the motif was lost in several phylogenetic lineages and replaced with another motif or an alternative mechanism of telomere maintenance (Frydrychová & Marec, 2002; Frydrychová et al., 2004; Mason, Randall, & Capkova Frydrychova, 2016; Mravinac, Meštrović, Cavrak, & Plohl, 2011).

Besides keeping chromosome integrity, telomeres are involved in chromosome pairing during meiosis and telomere–telomere sister chromatid cohesion during mitotic anaphase as found in very different organisms (Antoniacci & Skibbens, 2006; Carlton & Cande, 2002; Danjinou et al., 1999; Lee, Conrad, & Dresser, 2012; Rockmill & Roeder, 1998). Studies in several vertebrate species also suggest a potential role of telomeric repeats in karyotype evolution through additional intrachromosomal sites, the so‐called interstitial telomeric sequences (ITSs) (Bruschi, Rivera, Lima, Zúñiga, & Recco‐Pimentel, 2014; Meyne et al., 1990). In some species, the occurrence of ITS can be correlated with the evolutionary changes of karyotypes due to telomere–telomere fusions of the chromosomes, intrachromosomal rearrangements (inversions), unequal crossing over, or the insertion of telomeric DNA into unstable sites during the repair of double‐strand breaks (Bolzán & Bianchi, 2006; Lin & Yan, 2008; Meyne et al., 1990). In insects, ITSs consisting of the (TTAGG)_*n*_ motif were so far identified only in a species with holokinetic chromosomes, the vapourer moth *Orgiya antiqua* (Linnaeus) (Rego & Marec, 2003). This species has a reduced chromosome number, and the observed ITSs most probably reflect remnants of multiple chromosome fusions of ancestral chromosomes.

**Figure 1:**
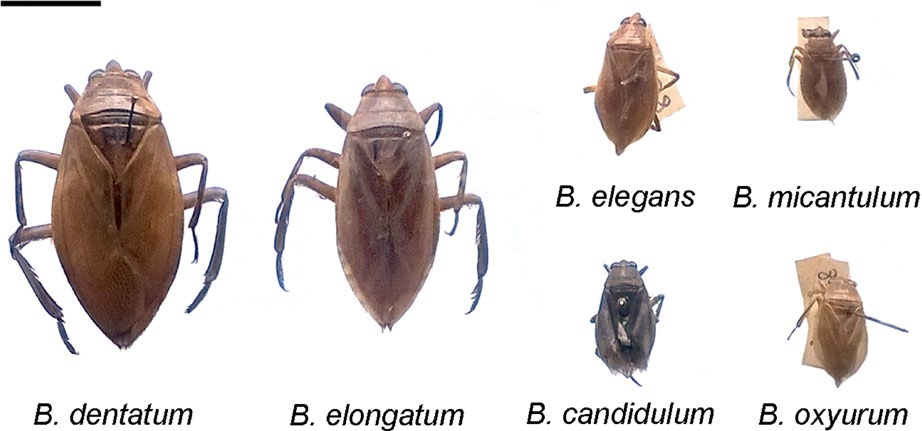
*Belostoma* giant water bugs from Argentina cytogenetically analyzed. Bar = 1 cm

The giant water bugs Belostomatidae play an important role as biological agents in freshwater ecosystems because they are intermediate‐stage predators in the food chain of their communities and are useful in the control of the most efficient vector species for malaria and dengue transmission, *Aedes* and *Anopheles*, given that they feed effectively on their larvae and pupae (Kweka et al., 2011; Saha, Aditya, Bal, & Saha, 2007; Schaefer & Panizzi, 2000; Sivagnaname, 2009). In the genus *Belostoma* (Heteroptera, Belostomatidae), previous cytogenetic studies showed that 17 species differ from one another in chromosome number, sex chromosome system, and several other chromosomal characters (Bozini Gallo et al., 2017; Chirino & Bressa, 2014; Chirino, Papeschi, & Bressa, 2013; Papeschi & Bidau, 1985; Papeschi & Bressa, 2006). This genus is the most diverse by including 61 species mainly distributed from Colombia and Brazil to Argentina and Chile (Heckman, 2011; Polhemus & Polhemus, 2008; Ribeiro & Estévez, 2009; Schnack, 1976). However, species delimitation is difficult due to they are very similar in coloration and appearance, only males or rarely only females can be identified, and there is no efficient key (Figure 1). Besides, it was also found out that Argentinean and Brazilian allopatric populations of both *B. candidulum* Montandon and *B. cummingsi* De Carlo, which are geographically separated by long distances and are restricted to small geographic areas (Ribeiro, 2007; Ribeiro & Estévez, 2009), should be considered as chromosomal races or cryptic species by having different chromosome complements (Bozini Gallo et al., 2017; Chirino & Bressa, 2014; Papeschi & Bidau, 1985). In *Belostoma*, it has been proposed that the ancestral chromosome number of 2n = 26 + XY/XX (male/female) changed during speciation by fragmentation of the X chromosome, resulting in a multiple sex chromosome system and the male karyotype of 2n = 26 + X_1_X_2_Y while preserving the ancestral pair of NOR–autosomes. Alternatively, several autosome–autosome fusions and a fusion between the ancestral sex chromosome pair and the pair of NOR–autosomes led to reduced chromosome numbers (2n = 14 + XY, 2n = 12 + XY, 2n = 6 + XY) and the increase in chromosome size. The fusion of sex chromosomes with both NOR–autosomes has been demonstrated by the presence of major ribosomal DNA (rDNA) clusters in both X and Y chromosomes (Chirino & Bressa, 2014; Chirino et al., 2013; Papeschi & Bressa, 2006). In male meiosis of all *Belostoma* species studied, at least one chiasma per bivalent is found, which is thought to be necessary for the regular segregation of homologs to opposite poles during meiosis I. The terminal/subterminal end‐to‐end connections between homologs facilitate their recombination and help to align them at metaphase plate, and thus ensure the pole‐to‐pole orientation of homologous chromosomes (Chirino & Bressa, 2014; Chirino et al., 2013; Papeschi, 1988; Papeschi & Bidau, 1985; Papeschi & Bressa, 2006). Hence, one could argue that the telomeres are hot spots of pairing and recombination as they are restricted to chromosome ends.

To further explore the karyotype evolution in giant water bugs, we examined the presence and distribution of the TTAGG telomeric repeats and ITS in *B. candidulum* (12 + XY/XX; male/female), *B. dentatum* (Mayr) (26 + X_1_X_2_Y/X_1_X_1_X_2_X_2_), *B. elegans* (Mayr) (26 + X_1_X_2_Y/X_1_X_1_X_2_X_2_), *B. elongatum* Montandon (26 + X_1_X_2_Y/X_1_X_1_X_2_X_2_), *B. micantulum* (Stål) (14 + XY/XX), and *B. oxyurum* (Dufour) (6 + XY/XX), by fluorescence in situ hybridization (FISH) with (TTAGG)_*n*_ probes. The results obtained clearly support the hypothesis that the remarkable karyotype variability of *Belostoma* species was mainly caused by telomere–telomere fusions of the holokinetic chromosomes, which differentiated the karyotypes of extant species from a common ancestor with 2n = 26 + XY/XX.

## 2 MATERIALS AND METHODS

### 2.1 Insects

Fifteen specimens, belonging to six species of *Belostoma*, were collected in different provinces of Argentina and taxonomically determined (Ribeiro & Estévez, 2009; Schnack, 1976) (Table 1).

**Table 1:** Species of *Belostoma* used in this study including the number and gender of adults examined, diploid chromosome number (2n), and collection sites

Species	Specimens	2n	Locality (province) in Argentina	Geographical coordinates
*B. candidulum* a	2 males	14 + XY	El Palmar National Park (Entre Ríos)	31°52′49″S, 58°19′30″W
	1 male	14 + XY	Corrientes (Corrientes)	27°28′16″S, 58°50′22″W
*B. dentatum*	1 female	26 + X_1_X_1_X_2_X_2_	Corrientes (Corrientes)	27°28′16″S, 58°50′22″W
*B. elegans*	1 female	26 + X_1_X_1_X_2_X_2_	Saavedra Park, La Plata (Buenos Aires)	34°55′53″S, 57°56′27″W
	1 male	26 + X_1_X_2_Y	Corrientes (Corrientes)	27°28′16″S, 58°50′22″W
	1 male	26 + X_1_X_2_Y	University City (Buenos Aires city)	34°32′32″S, 58°26′38″W
*B. elongatum*	1 male	26 + X_1_X_2_Y	Otamendi Nature Reserve (Buenos Aires)	34°14′03″S, 58°53′10″W
	2 males	26 + X_1_X_2_Y	Ibera Nature Reserve (Corrientes)	28°16′27″S, 57°26′15″W
*B. micantulum*	1 male	14 + XY	Ibera Nature Reserve (Corrientes)	28°16′27″S, 57°26′15″W
	2 males	14 + XY	San Cristóbal (Santa Fe)	30°19′00″S, 61°14′00″W
*B. oxyurum*	2 males	6 + XY	Otamendi Nature Reserve (Buenos Aires)	34°14′03″S, 58°53′10″W

a Chromosomal race from Argentinean population (see Chirino & Bressa, 2014 for details).

### 2.2 Chromosome preparations

Specimens were brought to the laboratory alive and their gonads dissected out in a physiological solution, swollen in a hypotonic solution, and fixed (Chirino et al., 2013). Gonads were transferred into a drop of 60% acetic acid, and their cells were dissociated with the help of tungsten needles and spread on the slide using a heating plate at 45°C (Traut, 1976). The preparations were dehydrated in an ethanol series (70%, 80%, and 96%, 30 s each) and stored at −20°C until use.

### 2.3 Telomeric probes

Unlabeled (TTAGG)_*n*_ telomeric probes were generated by the nontemplate polymerase chain reaction (PCR) method (Ijdo, Baldini, Ward, & Reeders, 1991; Sahara et al., 1999). For FISH, probes were labeled by nick translation with biotin‐14‐dUTP using a BioNick Labeling System (Invitrogen, Life Technologies Inc., San Diego, CA, USA).

### 2.4 Fluorescence in situ hybridization (FISH)

Chromosome preparations were removed from freezer, dehydrated in an ethanol series, and air‐dried. The preparations were treated with 10 mmol/L HCl for 10 min at 37°C in shaking water bath to remove cytoplasm, washed three times in 2× SSC for 5 min each at RT, digested with 100 μg/ml RNase A (Sigma‐Aldrich, St. Louis, MO, USA) in 2× SSC for 60 min at 37°C in a humid chamber, and incubated in 5× Denhardt's solution (50× Denhardt is 1% Ficoll, 1% polyvinylpyrrolidone, 1% bovine serum albumin) for 30 min at 37°C (Sahara et al., 1999). Then, they were denatured in 70% deionized formamide for 3 min 30 s at 68°C, dehydrated in a cold ethanol series, and air‐dried.

For each slide, 10 μl of hybridization mixture containing 50 ng of the biotin‐labeled telomere probe, 10 μg of salmon sperm DNA (Sigma‐Aldrich), 70% deionized formamide, and 20% dextran sulfate in 2× SSC was used. The mixture was denatured for 5 min at 90°C and immediately chilled on ice for at least 3 min. After denaturation, 10 μl of the mixture was spotted on each slide, and the slides were incubated overnight at 37°C in a humid chamber. Posthybridization washes, detection of hybridization probe signals using Cy3‐conjugated streptavidin (Jackson ImmunoRes. Labs. Inc., West Grove, PA, USA), and one round of amplification with biotinylated antistreptavidin (Vector Labs. Inc., Burlingame, CA, USA) and Cy3‐conjugated streptavidin were performed (Sahara et al., 1999). The preparations were counterstained with 0.1 μg/ml 4′6‐diamidino‐2‐phenylindole (DAPI; Fluka BioChemika, Sigma‐Aldrich Production GmbH, Buchs, Switzerland) and mounted in antifade based on DABCO (Sigma‐Aldrich Production GmbH, Buchs, Switzerland).

### 2.5 Microscopy and image processing

Preparations were observed in a Leica DMLB epifluorescence microscope equipped with a Leica DFC350 FX CCD camera and Leica IM50 software, version 4.0 (Leica Microsystems Imaging Solutions Ltd., Cambridge, UK). Black‐and‐white images were recorded separately for each fluorescent dye. Images were pseudo‐colored (light blue for DAPI and red for Cy3) and processed with Adobe Photoshop CS6 version 6.1 (1999–2012) software (Adobe Systems Inc.).

### 2.6 Statistical analysis

The total chromosome length (TCL; mean ± *SE*) of all bivalents and sex chromosomes was measured with Micro Measure for Windows, version 3.3, in metaphase I. Differences in TCL among species were compared using the Kruskal–Wallis ANOVA test on ranks for global comparisons (*p *<* *.05), followed by Mann–Whitney U tests for contrasts between treatments, as the data were not normally distributed and were not homoscedastic (Daniel, 1990). Statistical analyses were performed using Statview software (SAS Institute, 1992).

## 3 RESULTS

**Figure 2:**
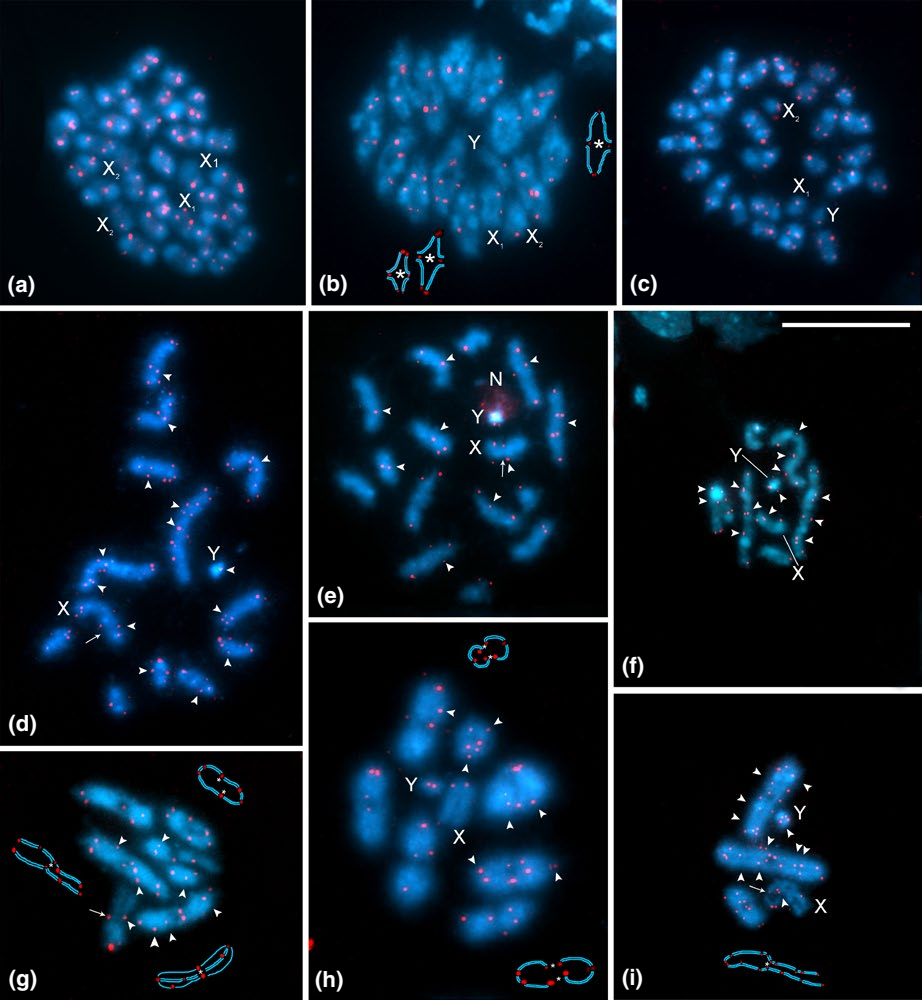
FISH of the (TTAGG)_*n*_ telomere probe (red signals) in mitotic and meiotic chromosomes of six species of *Belostoma* counterstained with DAPI (blue). (a) Oogonial metaphase of *B*. *dentatum*, 2n = 26 + X_1_X_1_X_2_X_2_. (b) Male diakinesis of *B. elegans*, 2n = 26 + X_1_X_2_Y, *n* = 13 + X_1_X_2_Y. (c) Spermatogonial metaphase of *B. elongatum*, 2n = 26 + X_1_X_2_Y. (d) Spermatogonial prometaphase of *B*. *candidulum*, 2n = 12 + XY. (e) Spermatogonial prometaphase of *B*. *micantulum*, 2n = 14 + XY. (f) Spermatogonial prometaphase of *B*. *oxyurum*, 2n = 6 + XY. (g) Male diakinesis of *B*. *candidulum*,* n* = 6 + XY. (h) Male diakinesis/metaphase I of *B*. *micantulum*,* n* = 7 + XY. (i) Male metaphase I of *B*. *oxyurum*,* n* = 3 + XY. N: nucleolus. X, Y: sex chromosomes. Arrows show the secondary constriction in the X chromosomes. Arrowheads indicate the interstitial hybridization signals. Asterisks in schematic drawings of selected bivalents in B, G, H, and I indicate positions of chiasmata and red dots positions of telomeric and ITS signals. Bar = 10 μm

FISH experiments with (TTAGG)_*n*_ probe showed twin hybridization signals at terminal and/or subterminal positions of the autosomes and sex chromosomes in all six *Belostoma* species (Figure 2). The telomeric signals were observed at different stages of mitosis in both sexes as well as in male meiosis. However, in some chromosome complements, not all chromosome ends showed hybridization signals.

In species with a high chromosome number (2n = 26 + X_1_X_2_Y/X_1_X_1_X_2_X_2_, male/female), namely *B. dentatum* (Figure 2a), *B. elegans* (Figure 2b), and *B. elongatum* (Figure 2c), the telomere probe hybridized to the ends of the chromosomes, without any interstitial hybridization signals (ITSs). In the karyotype of these species, the autosomes decrease gradually in size, the two negatively heteropycnotic X chromosomes differ slightly in size, and the Y chromosome is the smallest of the complement. On the other hand, *B. candidulum* (2n = 12 + XY; Figure 2d, g), *B. micantulum* (2n = 14 + XY; Figure 2e, h), and *B. oxyurum* (2n = 6 + XY; Figure 2f, i), the species with reduced chromosome numbers, exhibited hybridization signals in the inner parts of some chromosomes in addition to the typical telomeric signals, indicating the presence of ITS in both the autosomes and the sex chromosomes (Figure 2d‐h). The chromosomes displayed either single or double hybridization signals in both terminal/subterminal positions and, in some cells, different chromosomes per cell showed from one to four ITSs, indicating that sequences were the result of telomere–telomere fusions. In these species, the X chromosome showed a medial or subterminal constriction with ITS signals of the telomeric probe in its close proximity (Figure 2d‐g). This constriction did not colocalize with the nucleolus organizing region (NOR), which was located at terminal positions of both the X and Y chromosomes. During meiotic prophase I, a region with a different pycnosis and condensation could be distinguished from the constriction to the end of the X univalent (Figure 2 g, i). There were differences in the number of ITS per autosomal bivalent within each species and between the species. In *B. candidulum* and *B. micantulum*, from one to three pairs of ITS signals were observed in the largest autosomal bivalent and one pair of ITS signals in each of the other bivalents, except for the smallest bivalent of the set (1.13 ± 0.03 and 1.17 ± 0.02 ITS/cell for *B. candidulum* and *B. micantulum*, respectively; Figure 2d‐e, g, h). At metaphase I of *B. oxyurum*, two or three double or single ITS signals of the telomere probe were observed in the largest and in the second bivalents, and one in the smallest bivalent (2.19 ± 0.01 ITS/cell; Figure 2i). However, more interstitial signals were observed at mitotic prophase and pachytene stages than at metaphase I (Figure 2f, i). These discrepancies likely suggest that ITSs are present in a low copy number to be detected by standard FISH when the chromosomes are highly condensed. We also observed differences in telomere signal intensities. Both the telomeric and ITS signals were not always balanced between homologous chromosomes within the same cell and in the same chromosome. This variation could be partly due to the structure and condensation of chromatin and partly reflect the length of telomeric sequences.

In addition, we observed differences in TCL between the six species studied (Kruskal–Wallis ANOVA: *H*_5, 169_ = 130.19, *p *<* *.0001). Three species with high chromosome numbers showed a similar TCL (40.18 ± 0.88 μm in *B. dentatum*, 43.12 ± 0.85 μm in *B. elegans*, and 37.60 ± 0.55 μm in *B. elongatum*) but higher than the species with reduced chromosome numbers. *Belostoma candidulum* (28.59 ± 0.55 μm) and *B. micantulum* (26.66 ± 1.15 μm) exhibited a higher TCL than *B. oxyurum* (20.83 ± 0.34 μm). Thus, two inverse relationships are evident: between the chromosome number and chromosome size and between the presence and/or absence of interstitial signals and chromosome number (Figure 2).

## 4 DISCUSSION

In this study, we mapped telomeric and ITS repeats in six species of *Belostoma* using FISH with (TTAGG)_*n*_ probes. Our results clearly showed that the (TTAGG)_*n*_ motif is a component of the telomeres in *Belostoma dentatum*,* B. elegans*,* B. elongatum*,* B. candidulum*,* B. micantulum*, and *B. oxyurum*. This motif is widespread through different lineages of insects and other arthropods, and it is considered as the ancestral sequence of telomeres in chromosomes of arthropods (Traut et al., 2007; Vítková et al., 2005). However, insects are a heterogeneous group for the presence or absence of the (TTAGG)_*n*_ telomeric sequence (Frydrychová et al., 2004). This also applies to Hemiptera, where the TTAGG repeats were initially reported only for aphids, coccids (Sternorrhyncha), and leafhoppers (Auchenorrhyncha) (Bizzaro, Mandrioli, Zanotti, Giusti, & Manicardi, 2000; Frydrychová et al., 2004; Golub, Kuznetsova, & Rakitov, 2014; Kuznetsova, Maryańska‐Nadachowska, Anokhin, & Aguin‐Pombo, 2015; Maryańska‐Nadachowska, Anokhin, Gnezdilov, & Kuznetsova, 2016; Spence, Blackman, Testa, & Ready, 1998), but not for seven true bug species (Heteroptera), *Halyomorpha mista* (Uhler), *Pyrrhocoris apterus* (Linnaeus), *Eurydema oleracea* (Linnaeus), and *Graphosoma lineatum* (Linnaeus) from the clade Pentatomomorpha, and *Deraeocoris rutilus* (Herrich & Schäffer), *Megaloceroea recticornis* (Geoffroy), and *Cimex lectularius* (Linnaeus) from the clade Cimicomorpha (Grozeva, Kuznetsova, & Anokhin, 2011; Okazaki et al., 1993; Sahara et al., 1999). Nevertheless, the presence of (TTAGG)_*n*_ motif was recently confirmed in the telomeres of many phylogenetically distant Auchenorrhyncha species, the mealybug *Planococcus lilacinus* (Cockerell) (Sternorrhyncha), the true bug *Lethocerus patruelis* (Stål) (Heteroptera, Belostomatidae), the kissing bugs *Triatoma infestans* (Klug), *T. dimidiata* (Latreille), *Dipetalogaster maxima* Uhler, and *Rhodnius prolixus* Stål (Heteroptera, Triatominae), and even the moss bug *Peloridium pomponorum* Shcherbakov, belonging to Coleorrhyncha, the sister group of Heteroptera (Golub et al., 2014; Kuznetsova, Grozeva, & Anokhin, 2012; Kuznetsova, Grozeva, Hartung, & Anokhin, 2015; Kuznetsova, Maryańska‐Nadachowska, et al., 2015; Maryańska‐Nadachowska, Ksuznetsova, & Karamysheva, 2013; Maryańska‐Nadachowska et al., 2016; Mohan, Rani, Kulashreshta, & Kadandale, 2011; Pita et al., 2016). These findings together with the results of this study reinforce the hypothesis that the plesiomorphic (TTAGG)_*n*_ telomere structure is preserved in the heteropteran clade Nepomorpha.

The giant water bugs of the genus *Belostoma*, examined in this study, showed differences in the number, intensity, and position of hybridization signals of the (TTAGG)_*n*_ probes. Variation in the number and intensity of signals observed within chromosome complements of the same individual and between individuals of the same species could result from differences in the length of target TTAGG sequences and/or differences in hybridization efficiencies of FISH experiments performed. Nonetheless, these results together with previously published cytogenetic data (Chirino & Bressa, 2014; Chirino et al., 2013; Papeschi, 1988, 1994, 1996; Papeschi & Bidau, 1985; Papeschi & Bressa, 2006) support a hypothesis that the karyotype evolution in *Belostoma* species proceeded through fragmentation of the ancestral X chromosome and several autosome and/or autosome–sex chromosome fusions. In species with the modal diploid number of autosomes (26) and the multiple sex chromosome system (X_1_X_2_Y/X_1_X_1_X_2_X_2_), only true telomeric signals were found at the ends of chromosomes. However, in species with reduced autosome numbers (14, 12, 6) and the XY/XX sex chromosome system, several sites with ITS were identified in addition to the terminal telomeric sequences.

**Figure 3:**
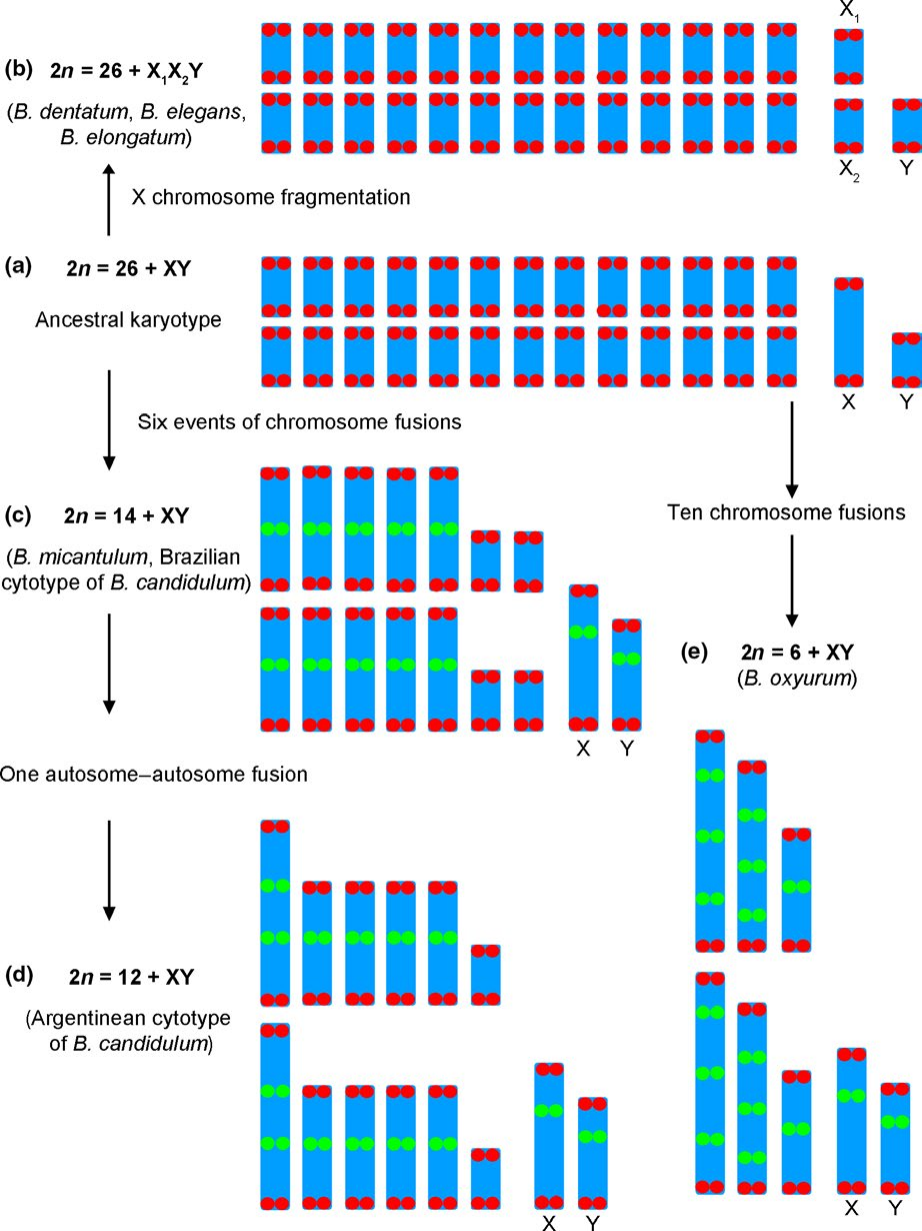
Hypothetical pattern of the karyotype evolution in the genus *Belostoma*. (a) Ancestral diploid karyotype (2n = 26 + XY/XX). (b) Karyotype of *B. dentatum*,* B. elegans,* and *B. elongatum* (2n = 26 + X_1_X_2_Y/X_1_X_1_X_2_X_2_). (c) Karyotype of *B. micantulum* and *B. candidulum* from Brazil (2n = 14 + XY/XX). (d) Karyotype of *B. candidulum* from Argentina (2n = 12 + XY/XX). (e) Karyotype of *B. oxyurum* (2n = 6 + XY/XX). Interstitial telomeric sequences (ITS, green) detected by FISH represent remnants of true telomeres (red) as a result of end‐to‐end chromosome fusion

The presence of ITS in *B. micantulum*,* B. candidulum*, and *B. oxyurum* chromosomes as well as differences in the number of hybridization signals detected by FISH suggest the origin of their karyotypes by means of several telomere–telomere fusions of chromosomes of the ancestral karyotype (2n = 26 + XY/XX) (Figure 3). Thus, the *B. micantulum* karyotype (2n = 14 + XY/XX) probably originated by six telomere–telomere fusions of the ancestral chromosomes, five of them between autosomal pairs, and one between an autosomal pair and the sex chromosome pair. Correspondingly, six pairs of chromosomes showed ITSs and two pairs were without ITS (Figures 2e and 3c). A similar mechanism of telomere–telomere fusions could be involved in the origin of *B. candidulum* from Brazil (2n = 16) (Figure 3d; revised in Chirino & Bressa, 2014). In accordance with this hypothesis, the *B. candidulum* karyotype from Argentina (2n = 12 + XY/XX) can be explained by an extra fusion between two pairs of autosomes, resulting in a pair of very large autosomes which bear two ITS sites each (Figures 2d and 3d). Finally, the karyotype of *B. oxyurum* (2n = 6 + XY/XX) could originate by a total of ten fusions, resulting in the largest pair of autosomes with three ITS sites, the second pair with three ITS sites, the third pair with only one ITS site, and the sex chromosome pair with one ITS (Figures 2f and 3e). Therefore, the most parsimonious mechanism could be the independent fusion in tandem of chromosomes of ancestral species leading to the observed karyotypes in the analyzed species of *Belostoma*.

Discussions on karyotype evolution in Heteroptera make use of the concept of modal numbers at level of family, tribe, or genera to propose the ancestral number for the analyzed group (Manna, 1984; Papeschi & Bressa, 2006; Ueshima, 1979). From a cytogenetic point of view, Nepomorpha may be regarded as containing five superfamilies: Nepoidea (2n = 4–46, modal number 2n = 28), Ochteroidea (2n = 35), Naucoroidea (2n = 20–51, modal number 2n = 28), Corixoidea (2n = 24), and Notonectoidea (2n = 23–26) (Chirino & Bressa, 2014; Chirino et al., 2013; Manna, 1984; Papeschi & Bressa, 2006; Ueshima, 1979; Wisoram, Saengthong, & Ngernsiri, 2013). In Nepoidea, the diploid chromosome number varies from 22 to 46 in Nepidae, with a modal number 2n = 43, and from 4 to 30 in Belostomatidae, being 2n = 28 the modal number. Besides, the XY/XX is the most common sex chromosome system because it has been described in more than 70% of heteropteran species studied. Within Belostomatidae, the previous cytogenetic studies in *Belostoma*,* Diplonychus,* and *Lethocerus* species revealed that the most frequent chromosome complement is 2n = 28 with the simple system XY/XX. Therefore, it is safe to assume that this chromosome number could be the ancestral complement of the family, especially considering that it occurs in the three genera. From this ancestral complement, all karyotypes of the extant species could arise through fusions and/or fragmentations of the autosomes and the sex chromosomes. The possibility of their occurrence is supported by the fact that the autosome and autosome–sex chromosome fusions have been found in natural populations of *B. plebejum* (Stål) from Argentina, allopatric populations of *B. cummingsi* and *B. candidulum* from Argentina and Brazil, and a sample of *Lethocerus indicus* (Lepeletier & Serville) from South‐East Asia characterized by having a neo‐XY sex chromosome system (Bozini Gallo et al., 2017; Chirino & Bressa, 2014; Papeschi, 1994; Wisoram et al., 2013). In turn, this evolutionary trend is strengthened by the existence of an inverse relationship between the chromosome size and the chromosome number (Chirino & Bressa, 2014; Papeschi, 1988, 1992). On the other hand, it is generally accepted that multiple sex chromosome systems in Heteroptera are the result of fragmentation(s) of the X and/or Y chromosome(s) from an ancestral simple system (see all references included in Chirino et al., 2013). Thus, the fragmentation of the ancestral X chromosome could have originated the multiple systems X_n_Y in *Belostoma* species, without implies a reduction in autosome number.

In a recent study, Bozini Gallo et al. (2017) proposed a new evolutionary hypothesis for *Belostoma* group, claiming that several karyotypes were originated through agmatoploidy (fragmentations), simploidy (fusions), heterochromatinization, and movement of 18S rDNA from an ancestral karyotype which had a low diploid number and a simple sex chromosome system. That hypothesis was suggested considering only eight species from Brazil without contemplating the global cytogenetic data previously reported from the Argentinean *Belostoma* species which possess reduced chromosome numbers, *Lethocerus* and *Diplonychus* species from other places, and other families and superfamilies belonging to the Nepomorpha clade. Taken together data previously published on Belostomatidae and other families of Nepomorpha along with the results here obtained, we consider that it is essential to take into account different chromosome features and cytological markers, which they must be analyzed simultaneously at different clade levels, for the purpose of determining the evolutionary trends in karyotype evolution discussions.

On the other hand, in *B. candidulum*,* B. micantulum*, and *B. oxyurum*, telomeric FISH revealed a heteromorphism in the number of ITS signals between homologs of the largest autosome pair. Some of these ITS signals might represent insertions of telomeric repeats that occurred during the repair of double‐stranded DNA breaks (Bolzán & Bianchi, 2006; Nergadze, Santagostino, Salzano, Mondello, & Giulotto, 2007). On the other hand, ITSs are believed to be remnants of heterochromatin that after chromosomal fusions may expand by a variety of amplification mechanisms, including transposition (Meyne et al., 1990; Nergadze et al., 2007). The presence of C‐positive bands terminally located in all *Belostoma* species (Chirino et al., 2013; Papeschi, 1988) supports the possible heterochromatin origin of ITS and the existence of polymorphic variants by amplification. These telomere‐like DNA sequences cannot be distinguished from the normal telomeric sequences by most conventional techniques. Finally, in telomere–telomere fusions, chromosome breakage could place in different part of the terminal region and involve all telomere sequences, part of it, or only the heterochromatic terminal region of the chromosomes (Meyne et al., 1990). Therefore, we can observe different hybridization patterns in each homologous of the largest autosomal pair.

As telomeres are required for maintaining chromosome stability and integrity (Blackburn, 1991; Fajkus et al., 2005; de Lange, 2004; Louis & Vershinin, 2005), a prerequisite for the formation of telomere–telomere fusions should be either elimination or inactivation of telomeres. Therefore, terminal chromosomal fusions imply that the interstitial telomere sequences became dysfunctional. Epigenetic modification of DNA is likely the factor that confers the stability of ITS in *Belostoma* because these repetitive sequences could be hypermethylated, leading to the protection of the chromosomal integrity by gene disruption, repressing recombination, and silencing of neighboring gene replication (Benetti, García‐Cao, & Blasco, 2007; Gonzalo et al., 2006). However, we cannot exclude other options, such as amplification that would lead to different patterns of ITS between homologous chromosomes, deletion that would lead to the absence of ITS in some homologs, and substitution that would produce several hundred base pairs of tandem repeats with many degenerate units (Bolzán & Bianchi, 2006; Danjinou et al., 1999; Fajkus et al., 2005; Lin & Yan, 2008).

In summary, *Belostoma* constitutes a very interesting group from a cytogenetic point of view, as it exhibits a great variety of chromosome complements with simple and multiple sex chromosome systems. The procedures applied here provide very valuable cytogenetic markers to compare karyotypes of phylogenetically related species. They may also contribute to a better understanding of taxonomic relationships and elucidate the high plasticity of nuclear genomes at the chromosomal level and the potential for genome modification in the course of the speciation processes.

## CONFLICT OF INTEREST

None declared.
